# A MITE-based genotyping method to reveal hundreds of DNA polymorphisms in an animal genome after a few generations of artificial selection

**DOI:** 10.1186/1471-2164-9-459

**Published:** 2008-10-06

**Authors:** Aurélie Bonin, Margot Paris, Laurence Després, Guillaume Tetreau, Jean-Philippe David, Andrzej Kilian

**Affiliations:** 1 Laboratoire d'Ecologie Alpine, CNRS-UMR 5553, Université Joseph Fourier, BP 53, 38041 Grenoble cedex 09, France; 2 Diversity Arrays Technology P/L, PO Box 7141, Yarralumla, ACT 2600, Australia

## Abstract

**Background:**

For most organisms, developing hundreds of genetic markers spanning the whole genome still requires excessive if not unrealistic efforts. In this context, there is an obvious need for methodologies allowing the low-cost, fast and high-throughput genotyping of virtually any species, such as the Diversity Arrays Technology (DArT). One of the crucial steps of the DArT technique is the genome complexity reduction, which allows obtaining a genomic representation characteristic of the studied DNA sample and necessary for subsequent genotyping. In this article, using the mosquito *Aedes aegypti *as a study model, we describe a new genome complexity reduction method taking advantage of the abundance of miniature inverted repeat transposable elements (MITEs) in the genome of this species.

**Results:**

*Ae. aegypti *genomic representations were produced following a two-step procedure: (1) restriction digestion of the genomic DNA and simultaneous ligation of a specific adaptor to compatible ends, and (2) amplification of restriction fragments containing a particular MITE element called *Pony *using two primers, one annealing to the adaptor sequence and one annealing to a conserved sequence motif of the *Pony *element. Using this protocol, we constructed a library comprising more than 6,000 DArT clones, of which at least 5.70% were highly reliable polymorphic markers for two closely related mosquito strains separated by only a few generations of artificial selection. Within this dataset, linkage disequilibrium was low, and marker redundancy was evaluated at 2.86% only. Most of the detected genetic variability was observed between the two studied mosquito strains, but individuals of the same strain could still be clearly distinguished.

**Conclusion:**

The new complexity reduction method was particularly efficient to reveal genetic polymorphisms in *Ae. egypti*. Overall, our results testify of the flexibility of the DArT genotyping technique and open new prospects as regards its application to a wider range of species, including animals which have been refractory to it so far. DArT has also a role to play in the current burst of whole-genome scans carried out in various organisms, which track signatures of selection in order to unravel the basis of genetic adaptation.

## Background

Since the early sixties and the first protein gels to assess genetic diversity in human and *Drosophila*, genotyping methods have gone a very long way. Researchers working on model organisms have now at their disposal a repertoire of different molecular markers to help answer various biological questions [1]. For such species, the recent advances in genotyping throughputs and data management allow to simultaneously examine many loci in the genome of many individuals, leading the way to the genomic era [2]. However, as regards non-model species, the picture is not so bright. For most organisms indeed, whole genome surveys are often hampered by a shortage of genomic sequences and/or a lack of interspecific transferability of known molecular markers such as microsatellites or single nucleotide polymorphisms (SNPs) [3,4]. In this context, there is an obvious need for new methodologies allowing the low-cost, fast and high-throughput genotyping of virtually any species.

The Diversity Arrays Technology (DArT) has the potential to fill in this gap [5]. This innovative genotyping method can provide from hundreds to tens of thousands of highly reliable markers for any species in theory, as it does not require any precise information about the genome sequence [5,6]. Moreover, DArT was recently shown to provide good genome coverage in wheat and barley [6,7]. The keystone of the DArT protocol is a step called "genome complexity reduction". This step aims at providing a genomic representation of the studied DNA sample, by extracting informative loci while avoiding repetitive sequences that usually plague eukaryote genomes. This is generally achieved by methylation sensitive restriction enzyme digestion, adaptor ligation and subsequent PCR amplification [6]. The number of markers DArT detects is determined primarily by the level of DNA sequence variation in the material subjected to analysis and by the complexity reduction method deployed [8]. In many cultivated species in which selection through traditional and modern breeding reduced genetic diversity, DArT usually generates several hundreds highly reproducible markers in a single assay in a single biparental cross [6]. Another noteworthy property of DArT markers is that their sequence is easily accessible. This distinguishes them from other random markers such as amplified fragment length polymorphisms (AFLPs) and offers interesting perspectives in functional genomics. Overall, these characteristics make DArT a method of choice for non-model species [9] when it comes to assess genetic variation at the genome scale, to construct quantitative trait loci (QTL) or linkage maps, or to conduct genomic scans in order to track loci under selection in the genome.

The DArT technique was applied for the first time to the rice genome [5]. Thereafter, it has met an increasing success and was developed for a wide range of crop and plant species [6,9-11] and was even used to identify soil micro-organisms [12]. However, despite an initial proof-of-concept work on mouse (Jaccoud, pers. comm.), attempts to develop DArTs for animals have been strongly delayed so far. This can be explained by differences in genome organization between plants and animals, demanding significant changes in the complexity reduction step.

This study has been motivated by research on the genetic basis of insecticide resistance in the mosquito *Aedes aegypti*, the primary vector species for the yellow fever and dengue viruses [13]. In particular, we were interested in characterizing genes linked to resistance to *Bacillus thuringiensis *var *israelensis *(*Bti*), a soil bacterium producing insecticidal crystal proteins which are widely used for controlling *Aedes *mosquito larvae [14]. In order to identify these genes, we chose to adopt a population genomics approach, i.e. to screen the genome of *Ae. aegypti *to detect loci showing a signature of selection by *Bti*. A prerequisite was thus to obtain many (several hundreds) random markers that could be surveyed at low cost and effort, and the DArT technology appeared as an appealing option for this purpose given the current shortage of SNPs markers isolated in *Ae. aegypti*.

In this article, we present a modification of the complexity reduction step of the DArT protocol taking advantage of the abundance of transposable elements (TEs) in many eukaryote genomes. Indeed, most TEs have conserved sequence motifs which can serve as specific anchors for the primers used to amplify fragments from the DArT genomic representation. Here, we implement the DArT technique for *Aedes aegypti *by targeting a TE called *Pony*, which belongs to the miniature inverted repeat transposable element (MITE) family of TEs and can be found in many copies in the genome of *Ae. aegypti *[15]. We show that this method is powerful enough to detect DNA polymorphisms even between populations separated by only a few generations of artificial selection. Beyond these promising results, this example testifies of the flexibility of the DArT technology and opens new prospects as regards its application to a wider range of species, including animals which have been refractory to it so far.

## Results

### Principle of the new complexity reduction method implemented

The DArT technique is based on the analysis of "genomic representations", which are simplified surrogates of the DNA samples of interest. Concretely, a genomic representation is a set of DNA fragments of various sizes and sequences which are characteristic of the studied sample and obtained through highly reproducible (and preferentially technically simple) methods. These methods are usually based on restriction digestion: genomic DNA is digested using one or several restriction enzymes, with simultaneous ligation of appropriate adaptors to the restriction fragments and subsequent amplification of fragments by PCR using the adapter and the restriction site as targets for primer annealing [6]. A suitable genomic representation typically includes 5,000–20,000 amplified fragments, i.e. a number low enough to ensure the reproducibility of the PCR reaction, but high enough to yield a reasonable number of polymorphic markers. Fragment sizes are ideally evenly distributed in a 100–1000 bp range, and representations showing distinct bands on agarose gel are avoided because these are presumably derived from repetitive genomic sequences and/or mitochondrial or chloroplast DNA.

**Figure 1:**
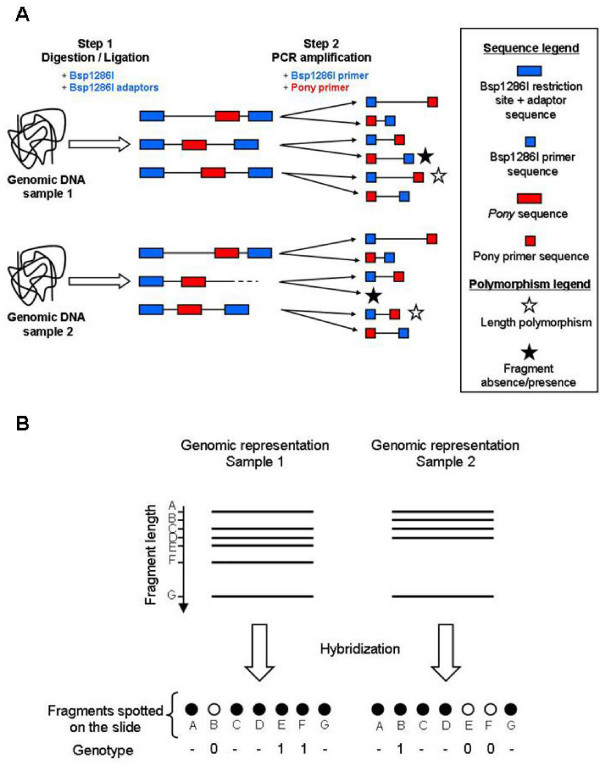
**Schematic illustration of the DArT protocol**. (**A**) Principle of the MITE-based genome complexity reduction method. Genomic DNA is digested by restriction enzyme Bsp1286I, and Bsp1286I adaptors are ligated to the generated overhangs. Then two rounds of PCR amplifications are performed using two primers: one annealing to Bsp1286I adaptors (Bsp1286I primer), and one complementary to a conserved sequence motif of the *Pony *element. For the most part, the resulting genomic representations include fragments with the Bsp1286I restriction site on one extremity and the *Pony *motif on the other one, because the PCR conditions are adjusted to preferably amplify this particular type of fragments. (**B**) Principle of the polymorphism detection on DArT microarrays. Genomic representations of each sample are hybridized against a library containing all fragments spotted on a slide. When a fragment is missing in one representation, it will not hybridize to the corresponding fragment on the slide. In this example, monomorphic fragments present in both representations are scored as '-' while polymorphic fragments present or absent in one representation are scored as '1' or '0', respectively.

In *Ae. aegypti*, several restriction enzyme combinations were tested (Additional file 1), but all of them gave genomic representations unfavourable to the application of the traditional DArT protocol, with clear repetitive bands and/or an unsuitable range size for the fragments (See Additional file 2 for an example of poor-quality genomic representations). On the basis of these results, a different strategy was thus adopted. The underlying idea was to exploit any kind of motifs occurring frequently in the genome as a second anchor during the PCR reaction, in addition to the adaptor-ligated restriction site. By adjusting PCR conditions, it was possible to preferably amplify fragments with the restriction site on one extremity and the chosen motif on the other one, so that the genomic representations to be obtained were expected to be a mixture of such fragments. Because of their abundance in eukaryote genomes, TEs were good candidates for such a purpose. We selected a particular MITE family named *Pony *to perform the role of second anchor in the *Ae. aegypti *genome (Figure 1). *Pony *TEs have all the characteristics of MITEs, including terminal inverted repeats, A+T richness and a small size [15]. Two highly divergent subfamilies, *Pony-A *and *Pony-B*, can be distinguished and occur in about 8,400 and 9,900 copies in *Ae. aegypti *genome, respectively [15]. We designed a primer targeting any *Pony *sequence present in the genome (PonyAll primer; Table 1), as well as one specific to the *Pony-B *subfamily (PonyB primer; Table 1).

**Table 1:** Adaptor and primer sequences used for preparation of genomic representations and library construction

	**Adaptors **(5'-3')	**Primers **(5'-3')
**Bsp1286I restriction site**		
	
*Preparation of genomic representations for genotyping*	*Bsp1286I adaptors I*	*Bsp1286I primer I*
	• Forward strand: equimolar mix of CATAGGTGTCCACAGTCGGTCTGCA CATAGGTGTCCACAGTCGGTCTGCT CATAGGTGTCCACAGTCGGTCTGCC	AGGTGTCCACAGTCGGTCT
	• Reverse strand GACCGACTGTGGAC	
	
*Library construction*	*Bsp1286I adaptors II*	*Bsp1286I primer II*
	• Forward strand: equimolar mix of CTGAGTAGTGCCAGAACGGTCTGCA CTGAGTAGTGCCAGAACGGTCTGCC CTGAGTAGTGCCAGAACGGTCTGCT	CTGAGTAGTGCCAGAACGGTC
	• Reverse strand GACCGTTCTGGCA	

***Pony *sequence**	-	**1536-clone preliminary library construction**
		*PonyAll primer*
		CGNAATNTGARYCAAAACGGTA
		**4608-clone expanded library construction and preparation of genomic representations for genotyping**
		*PonyB primer*
		GGANGCGTATTCTTYACCCAC

### Evaluation of the MITE display approach for DArT genotyping

A library comprising 6144 DArT clones was constructed using the approach described in Figure 1 (see *Methods *for details). Hybridizations were performed for 58 *Aedes aegypti *individuals with two, three and four replicated representations independently hybridized for 25, 4 and 29 individuals, respectively. These *Ae. aegypti *individuals belonged either to the Bora-Bora strain (29 individuals), susceptible to all insecticides, or to a strain artificially selected for several generations to develop resistance to the insecticidal *Bacillus thuringiensis *var *israelensis *(*Bti*) toxins (29 individuals; see *Methods*).

**Figure 2:**
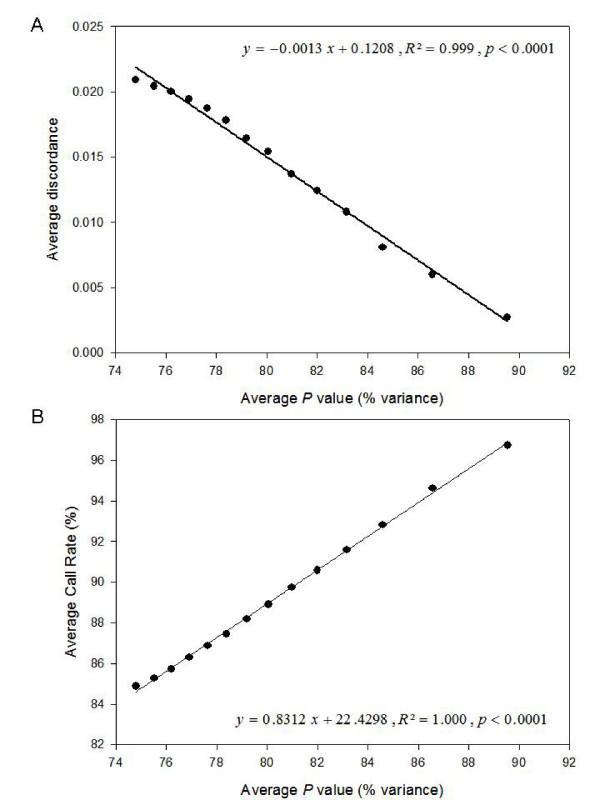
**Relationships between different quality parameters in the MITE library**. After discarding the most unreliable markers (discordance > 5%), remaining markers were sorted out by decreasing *P *values and grouped in bins with an increment of 50 markers between two successive bins. Within-group average *P *was plotted against within-group average discordance (**A**) and within-group average Call Rate (**B**).

The polymorphism analysis was performed on the obtained images focusing on three parameters which are central for the data quality: (1) the Call Rate, which corresponds to the percentage of successfully scored replicates for a given marker; (2) the *P *value, which measures the fraction of the total variation across all individuals due to bimodality (i.e. polymorphism) for a particular marker; and (3) the discordance, which measures the overall variation of scores within replicates and is thus an indication of the marker reproducibility. First, the most unreliable markers (discordance > 5%) were discarded from the analysis. The remaining markers were then sorted out by decreasing *P *values and grouped in bins with an increment of 50 markers between two successive bins. As shown in Figure 2A, the average discordance increased as the average *P *value of a marker group increased (Pearson correlation coefficient = -0.996, *p *< 0.01). There was also a quasi-linear relationship between the decrease in average *P *and the decrease in Call Rate (Pearson correlation coefficient = 0.999, *p *< 0.01; Figure 2B). Overall, the top 350 markers (*P *= 80.06%) showed a satisfactory Call Rate (88.90%) while displaying an acceptable level of discordance (1.48%). They met the standard quality thresholds usually applied to DArT data, giving a polymorphism rate of about 5.7% in the whole mosquito dataset.

### Working dataset and marker redundancy

The primary goal of this study was to characterize loci potentially responsible for resistance to *Bti*. For this purpose, population genomics was adopted to reveal loci displaying apparent selection footprints, such as an atypical pattern of genetic variability compared to the rest of the genome. To implement this approach, a robust estimation of the overall genetic diversity throughout the genome first had to be obtained. This was achieved by slightly relaxing the quality parameters to include more markers in our analyses and allow a more comprehensive sampling of the genome. A polymorphism analysis was thus performed with a minimum Call Rate, minimum *P *value, and maximum discordance set at 81%, 71%, and 5%, respectively. It identified a set of 476 markers (polymorphism rate = 7.75%) with a mean Call Rate, *P *value, and discordance of 87.72 %, 78.34 %, and 1.93%, respectively.

In this working dataset comprising 476 markers, linkage disequilibrium was low with only 0.72% and 1.90% of pairwise linkage indices > 0.95 and < 0.05, respectively. Furthermore, a subset of 70 markers involved in 76.92% of the marker pairs showing high linkage disequilibrium was sequenced. Only two of them (2.86%) turned out to be redundant, with one pair of markers differing by a gap and the second one by a mutation (similarity > 98.5% in BioEdit). After trimming the *Pony *motif, the 68 unique marker sequences (GenBank accession no. FJ231034–FJ231090; sequences shorter than 50 bp could not be deposited) were blasted against the *Ae. aegypti *genome. In total, 41 of them could be assigned without ambiguity to single genomic positions distributed on 40 different supercontigs. The two markers situated on the same supercontig were separated by more than 215 kb.

### Assessment of genetic diversity between and within mosquito strains

**Figure 3:**
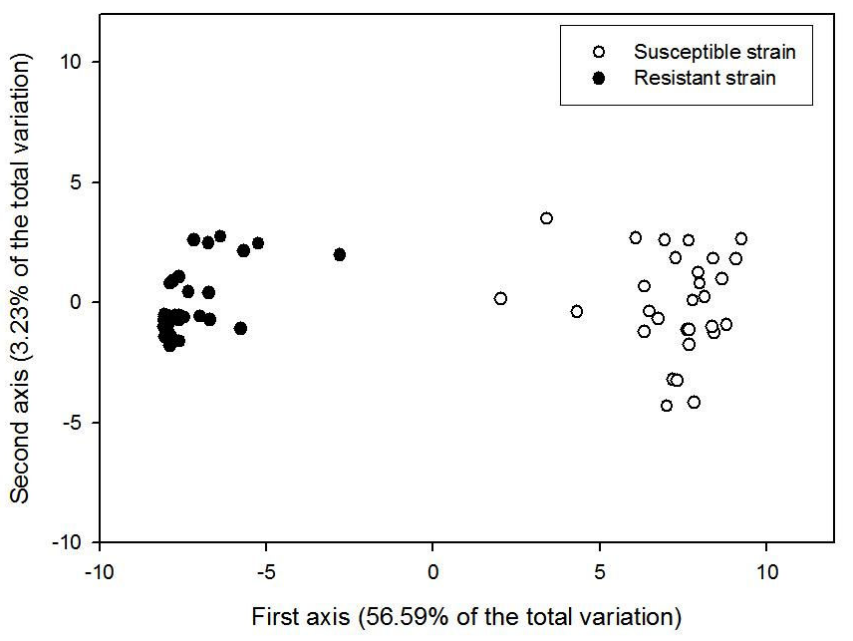
**Principal coordinate analysis**. A principal coordinate analysis (PCO) was carried out with the working dataset (476 DArT markers), and for each *Aedes aegypti *individual, the coordinate obtained for the first axis of the PCO was plotted against that obtained for the second axis.

The working dataset was used to assess the genetic diversity observed between and within the two studied mosquito strains. As revealed by an analysis of molecular variance (AMOVA), most of the genetic variation was distributed between the resistant and the susceptible strains (69.6 %, versus 30.4 % within strains; *p *< 0.0001 in both cases), corroborating the high genetic differentiation observed between the two strains (*Fst *= 0.556). Likewise, in a principal coordinate analysis (PCO), the first axis unmistakably separated the two strains and explained 56.59 % of the variation (Figure 3). However, when the PCO analysis was carried out on each strain independently, enough genetic diversity seemed to be retained within each strain to clearly differentiate individuals (data not shown), with the first two axes explaining 20.48 % and 26.72 % of the variation for the susceptible strain and the resistant strain, respectively.

Assessment of genetic diversity within strains allowed to complete this picture and revealed a strong difference between the two strains (Table 2). All the diversity indices calculated (Shannon index of phenotypic diversity S, mean Jaccard pair-wise coefficient, Nei's gene diversity and proportion of polymorphic markers at the 5% level, see *Methods *for more details) give evidence of a high level of genetic diversity within the susceptible strain, whereas these indices were substantially lower for the resistant strain, except the mean pairwise Jaccard coefficient.

**Table 2:** Indices of genetic diversity within each mosquito strain

**Diversity index**	**Susceptible strain**	**Resistant strain**
**Mean pairwise Jaccard coefficient (minimum – maximum)**	0.350 (0.196 – 0.643)	0.331 (0.110 – 0.590)

**Shannon index of phenotypic diversity S**	0.451	0.275

**Nei's gene diversity**	0.341	0.207

**Percentage of polymorphicmarkers at the 5% level**	94.1	58.6

## Discussion

In this article, we report a substantial refinement to the DArT genotyping technique which allowed its implementation for the yellow fever mosquito *Aedes aegypti*. More specifically, the genome complexity reduction step was achieved thanks to a MITE-display procedure which utilizes the *Ae. aegypti Pony *element [15] as an additional primer anchor. After restriction digestion of genomic DNA and ligation of specific adaptor to compatible ends, *Pony*-containing fragments were amplified using two primers, one annealing to the adaptor sequence and the other to a conserved sequence motif of the *Pony *element (Figure 1).

In the 6144-clone library we generated, the relationships observed between the quality parameters (Call Rate, *P *value and discordance) for the best markers were consistent with those reported in other species, for example wheat (see Figure 1 in [7]). It has to be noted that the mean discordance, although acceptable, is 3–5 times higher than that usually published in plants [6,7,11]. Genomic representations generated with the MITE procedure are potentially more complex, and there is also a competition for amplification between three types of fragments (i.e., "Restriction enzyme-Restriction enzyme" fragments, "Restriction enzyme-*Pony*" fragments, and "*Pony*-*Pony*" fragments). Both of these factors may contribute to increase the mean discordance in the MITE procedure compared to the traditional DArT protocol. In any case, the discordance parameter can be viewed as a genotyping error rate, and the value reported here (1.48%) is excellent in comparison to those typically obtained with other marker systems [16]. This high reproducibility of the DArT technique is mainly due to the routine practice of systematically genotyping samples at least twice, in order to discard unreliable markers as soon as the scoring step. Another reason of this high data quality is the computerized scoring of DArT markers, which transforms detected fluorescence intensities into presence/absence of a given fragment and limits scoring subjectivity.

Our MITE approach was successful to reveal a substantial number of DNA polymorphisms in the two closely related laboratory strains of *Ae. aegypti *studied here, with 5.70 % of highly reliable polymorphic markers in the library. This polymorphism rate is slightly lower than that obtained in other species where the MITE procedure has also been tested, probably because of an inherent lower diversity in the laboratory material studied here. In the sugarcane genome, for example, 9.78 % of the cloned fragments turned out to be polymorphic with similar average quality parameters (Heller-Uszynska *et al*., submitted).

Our working dataset, containing 476 polymorphic DArT markers selected with less stringent quality parameters, helped highlight substantial genetic differences between the two mosquito strains, with an observed *Fst *value reaching 0.556. Although the two strains diverged only 18 generations ago, this high level of genetic differentiation was not surprising in the light of the intensity of selection (80%) applied to the selected strain at each generation. In addition to this strong inter-strain genetic structure, the DArT dataset was also able to reveal high genetic diversity within both strains. Linkage analyses combined with marker sequencing also suggested that most of the markers were unique and scattered in the genome, and thus that our results could not be overly inflated by redundant or physically linked markers. In short, DArT markers appear to be discriminatory at both the intra- and inter-population levels, and have therefore the potential to become valuable tools in population genomics, even if they have not been used in this purpose so far.

As exemplifies by our study and others in molecular genotyping [17,18], TEs can be wonderful devices to help identify DNA polymorphisms. They are not only omnipresent in most genomes, but also tightly associated with various types of genetic variability, from changes in genome size and arrangement to single nucleotide mutations [19]. MITEs are particularly fascinating in this respect. The mode of transposition of most MITE families discovered so far has long remained mysterious [20,21]. They lack an active transposase, but according to recent studies, they seem to originate from ancestral autonomous TEs and to depend on transposases encoded by related autonomous TEs for contemporary transposition events [21,22]. Incidentally, there are hints indicating that stress may be a triggering factor in contemporary transposition events of MITEs [20]. In our case, one can speculate that the stress represented by insecticide selection could have played a role in shaping the strong genetic structure between the two strains. Moreover, despite their apparently deficient transposition capability, MITEs are usually present in high copy numbers in many eukaryote genomes. For example, the *Tourist *MITE superfamily represents alone more than 3 % of the rice genome [23], and between 10^3 ^and 10^4 ^copies of the *Angel *element can be found in the zebrafish genome [24]. MITEs also generally display well conserved motifs, which is particularly convenient for primer design [22]. Last but not least, they tend to insert in or near transcriptionally active genomic regions [20,21]. This particularity offers exciting prospects in studies of phenotypic traits of economical interest, or in genomic surveys tracking genes under selection. Interestingly, transposable elements often initiate the rapid evolution of insect resistance to insecticides, including resistance to *Bt *toxins [25,26]. In *Ae. aegypti*, DArT markers with atypically high genetic differentiation between the susceptible and resistant strains might thus be linked to genes involved in resistance to *Bti *toxins. Some of these markers have already been sequenced and at least two promising candidate genes have been identified in their vicinity (Paris, pers. comm.).

## Conclusion

The MITE approach described here (Figure 1) allowed obtaining the first DArT dataset ever published for an animal genome, although it has to be noted that the DArT method had previously been successfully developed for the mice genome (Jaccoud, pers. comm.). This example in *Ae. aegypti *testifies of the flexibility of the DArT genotyping technique, which can accommodate a wide range of other strategies for genome complexity reduction. Its quasi-universal applicability, the fact that limited genome information is necessary to its development, the possibility to obtain markers near coding regions and the possibility to rapidly sequence markers of interest are some of the features that can make DArT a serious competitor to other markers such as SNPs in non-model organisms [9]. In particular, DArT has a role to play in the current burst of whole-genome scans tracking signatures of selection in order to unravel the genetic basis of adaptation [2,27].

## Methods

### Biological material used and selection of the resistant strain

Two laboratory strains of *Ae. aegypti *were used as biological material in this study: the standard Bora-Bora strain, susceptible to all insecticides, and a strain artificially selected for several generations to develop resistance to a decomposed tree leaf litter showing a high toxicity when ingested by mosquito larvae. This toxic litter was proved to contain *Bacillus thuringiensis *var *israelensis *(*Bti*) bacteria strains from commercial origin [28]. *Bti *bacterium produces insecticidal toxins which are widely used for mosquito control, and the toxic litter was collected in a mosquito pond in Eastern France three months after treatment with commercial *Bti *insecticide (Bactimos, Valent Biosciences Corporation). This experimental design allowed us to study resistance mechanisms to *Bti *toxins in a situation close to field conditions.

Selection of the toxic leaf litter resistant strain was performed on early fourth-instar larvae of the Bora-Bora strain. At each generation, groups of 200 calibrated larvae were exposed to 30 mg of finely ground toxic leaf litter in 200 ml of tap water. Selection was carried out repeatedly for 18 generations, with an average of 2000 larvae being exposed to toxic litter per generation. At each generation, the experiment was stopped when mortality reached 80% in order to obtain a minimum of 300 adults for the next generation. The survivors were transferred to clean water, fed with hay pellets, and allowed to emerge as adults, reproduce, blood feed and lay eggs for the next generation. The average generation turnover was 30 days.

To monitor the evolution of resistance to toxic leaf litter, bioassays were conducted at each generation in plastic cups containing 20 fourth-instar larvae in 50 ml of tap water and various doses of toxic leaf litter [29]. The lethal dose for 50% of individuals after 24 h exposure (24 h-LD_50_) was determined using the Probit software [30]. The resistance ratio (RR) of the selected strain was calculated by dividing the 24 h-LD_50 _value of the selected strain with the value obtained for the susceptible strain. After 18 generations of selection, the RR of the resistant strain was 4-fold.

### Genomic DNA extraction

Genomic DNA was extracted from fourth-instar larvae using the Qiagen DNeasy Tissue Kit and protocol (Qiagen). To avoid bacterial contamination, the larvae midgut was removed carefully before DNA extraction.

### Preparation of genomic representations

For each sample, digestion and ligation reactions were carried out simultaneously at 37°C for 3 hours on 50 ng of genomic DNA, using 2 units of restriction enzyme Bsp1286I (New England Biolabs, NEB), 80 units of T4 DNA ligase (NEB) and 0.05 μM Bsp1286I adaptors I (Table 1), in a buffer with final concentrations of 10 mM Tris-OAc, 50 mM KOAc, 10 mM Mg(OAc)_2, _5 mM DTT (pH 7.8), 1 mM ATP and 100 ng/ml Bovine Serum Albumin (NEB). The obtained ligated products served as template in a first round of PCR amplification. For this purpose, ligated products were diluted five times with sterile water and 2.5 μl of the diluted product were added to a 22.5-μl PCR reaction mix leading to final concentrations of 0.04 μM of Bsp1286I primers I (Table 1), 0.4 μM of PonyB primers (Table 1), 10 mM Tris-Cl (pH 8.3), 50 mM KCl, 1.5 mM MgCl_2_, 0.1 mM of each dNTP and 1 unit of RedTaq DNA Polymerase (Sigma). The amplification reaction was performed with the following conditions: 94°C for 1 min; 20 cycles of 94°C for 30 sec, 50°C for 40 sec and 72°C for 1 min; followed by a final 7-min extension step at 72°C. The resulting PCR product was diluted 5 times with sterile water and 2.5 μl of the diluted product served as a template for a second round of amplification performed exactly as the first round except that the final volume was 50 μl, the final concentration of both primers and of each dNTP was 0.2 μM and 0.05 mM, respectively, and 2 units of RedTaq DNA polymerase were used.

### Construction of the DArT library and printing of microarrays

A preliminary 1536-clone library was first constructed based on genomic representations prepared for 29 individuals of each strain. These representations were obtained as described above, except the sequences of the adaptors and primers were slightly different (Bsp1286I adaptors II, Bsp1286I primer II and PonyAll primer; see Table 1), allowing the amplification from any type of *Pony *element (subfamily *A *or *B*). Representations were mixed according to the origin of the individuals to form a "susceptible pool" and a "resistant pool", and these pools were cloned separately in the PCR2.1 TOPO vector (TOPO TA Cloning kit, Invitrogen) following the manufacturer's instructions. Individual clones were grown overnight in 384-well plates containing LB medium with 100 μg/ml ampicillin and 4.4 % glycerol. Small aliquots of the cultures were used as templates for insert amplification in a 25-μl reaction containing 0.2 μM of each M13 forward and M13 reverse primers (Invitrogen), 50 μM of each dNTP, 50 mM Tris, 6 mM HCl, 16 mM (NH_4_)_2_SO_4_, 1.5 mM MgCl_2 _and 2 units of Taq Polymerase. The cycling conditions were as follows: 95°C for 4 min, 57°C for 35s, 72°C for 1 min followed by 35 cycles of 94°C for 35s, 52°C for 35s and 72°C for 1 min and final 72°C for 7 min. After amplification, PCR products were dried, washed with 70% ethanol and resuspended in a spotting buffer developed for poly-L-lysine coated microarray slides (Wenzl *et al*., in prep.). The final library contained 1536 clones, half of them originating from the "susceptible pool" and half of them from the "resistant pool", so that each pool of genetic diversity was equally represented.

The first genotyping experiments carried out with this preliminary library resulted in a low number of reliable polymorphic clones due to high levels of background noise in signal intensities (data not shown). One likely explanation was that the genomic representations hybridized against the library included too many fragments so that some of them were amplified stochastically during the two rounds of PCRs and/or did not hybridize specifically. To solve this problem, a second library was built, which was based on genomic representations with fewer fragments. It relied on the use of the PonyB primer designed to anneal only to the *Pony-B *sequences. Except for this different primer, the protocol was identical to that detailed above and lead to the production of a 4608-clone library. Clones from both libraries, i.e. 6144 in total, were printed in duplicates on poly-L-lysine-coated slides (Erie Scientific) using a *MicroGridII *arrayer (Biorobotics). Printed DNA spots were denatured and fixed on the surface of the slide by incubation in hot water (95°C) for 2 min, followed by dipping in 0.1 mM DTT, 0.1 mM EDTA solution and drying by centrifugation at 500 × g for 7 min.

### Individual genotyping using DArT microarrays

For each individual, at least two genomic representations were obtained independently as reported in the section "*Preparation of genomic representations*". Each representation was subsequently precipitated with one volume of isopropanol, washed with 70% ethanol and resuspended in 3.5 μl of sterile water. After a 3-min denaturation at 95°C, the representation was fluorescently-labelled for 3 hours at 37°C with 250 units of Klenow exo^- ^fragment of *E. coli *Polymerase I (NEB), 2.5 nmoles of either Cy3-dUTP or Cy5-dUTP (Amersham Bioscience) and 25 μM random decamers. A Cy3- and a Cy5-labelled samples (thereafter called targets) were combined one after the other to 60 μl of a hybridization buffer containing a 50:5:1 mixture of ExpressHyb (Clonetech), herring sperm DNA (Promega), FAM-labelled polylinker of the PCR2.1 vector (Invitrogen) used for library preparation, and 2 mM EDTA (pH 8.0). This mix was denaturated at 95°C for 3 min, deposited onto microarray slides and covered with a glass coverslip. Slides were incubated for 16 h in a humid chamber at 65°C. Following hybridization, coverslips were removed and slides were washed in 1 × SSC + 0.1% SDS for 5 min, 1 × SSC for 5 min, 0.2 × SSC for 2 min, and 0.02 × SSC for 30 sec, before being dried by centrifugation at 500 g for 7 min.

### Microarray scanning and data acquisition

A Tecan LS300 confocal laser scanner was used to scan the hybridized slides and generate three different TIF images per slide, one per type of hybridized dye (Cy3, Cy5 and FAM). Image and polymorphism analyses were performed with *DArTSoft *version 7.4.3, a software especially developed for this purpose by *Diversity Arrays Technology Pty. Ltd*. (Cayla *et al*., in prep.). Briefly, *DArTSoft *automatically localizes the arrayed spots on the images using a seeded-region-growth algorithm, rejects those with a weak reference signal, computes and normalizes background-subtracted relative hybridization intensities [e.g. log(cy3-target/FAM-reference)], and calculates the median value for replicate spots. Then, polymorphic clones are identified by means of a combination of ANOVA and fuzzy K-means clustering at a fuzziness level of 1.5 before being assigned as 'present' or 'absent' in each representation hybridized to the array.

### Linkage disequilibrium between markers and clone sequencing

Independence and uniqueness of DArT markers were evaluated by calculating the linkage index *I*_*k, l *_for each possible pair of markers *k *and *l*, according to the following formula:

$$I_{k,l}=\frac{1}{n}\sum \left|m_{ki}-m_{li}\right|$$

where *n *is the number of individuals, *m*_*ki *_the score (0/1) of individual *i *at marker *k*, and *m*_*li *_the score of individual *i *at marker *l*. Values of *I *< 0.05 or *I *> 0.95 are indicative of a statistical linkage disequilibrium between the two markers under consideration.

A subset of markers involved in pairs showing high linkage disequilibrium was selected and sequenced to assess the level of marker redundancy in the dataset. For these markers, bacterial cultures were sent to Genome Express^® (^) for insert amplification and sequencing with M13 forward and M13 reverse primers. Raw sequence files were trimmed and aligned using Bioedit 7.0.9 (). Marker sequences were also blasted against the full genomic sequence of *Ae. aegypti *(available for download at  and comprising 4758 supercontigs in total).

### Evaluation of genetic diversity between and within mosquito strains

Genetic variation was assessed within each mosquito strain by computing the Shannon index of phenotypic diversity S [31] with Popgene v1.32 () as well as the pair-wise Jaccard coefficients [32] with the vegdist function of the vegan R package  (). These two diversity indices do not rely on the estimation of allelic frequencies, which for dominant data such as DArT data requires additional assumptions (e.g. Hardy-Weinberg equilibrium). In addition, estimates of allelic frequencies were obtained with the Bayesian method with non-uniform prior distribution [33] implemented in AFLP-SURV v1.0 () [34], and used to calculate Nei's gene diversity [35] and the proportion of polymorphic markers at the 5% level within each strain. Genetic differentiation between strains was estimated by performing an analysis of molecular variance (AMOVA) using Arlequin v3.11 () [36] and by calculating the Fst index with AFLP-SURV v1.0. Principal coordinate analyses (PCO) were carried out with PCO v1.0 ().

## Abbreviations

*Bti*: *Bacillus thuringiensis *var *israelensis*; AFLP: amplified fragment length polymorphism; DArT: Diversity Arrays Technology; MITE: miniature inverted repeat transposable element; TE: transposable element; SNP: single nucleotide polymorphism.

## Authors' contributions

**AB **carried out the DArT experiments, analysed the data and drafted the manuscript. **MP **was in charge of the mosquito rearing, the bioessays, the DNA extractions and the sequence analyses, and wrote parts of the draft.** LD **and **JPD **conceived the overall study and helped with the writing. **GT **took part to the sequence analyses. **AK **designed the MITE protocol, coordinated the data analysis and revised the manuscript. All authors read and approved the final manuscript.

## Supplementary Material

Additional file 1 **Enzyme combinations tested to implement the traditional DArT protocol on the genome of *Aedes aegypti*.** Click here for file

Additional file 2 **Example of a poor-quality genomic representation obtained with enzyme combination PstI + Tsp509I.** Click here for file
