# Supplementary material for: A MITE-based genotyping method to reveal hundreds of DNA polymorphisms in an animal genome after a few generations of artificial selection
Source: BMC Genomics. 2008 Oct 6;9:459. doi: 10.1186/1471-2164-9-459 (PMC2579443; doi:10.1186/1471-2164-9-459)
Supplement: Additional file 1 — Enzyme combinations tested to implement the traditional DArT protocol on the genome of Aedes aegypti. [file 1471-2164-9-459-S1.doc]

## Additional file 1 - Enzyme combinations tested to implement the traditional DArT protocol on the genome of *Aedes aegypti*

| **Complexity reduction method** | **Enzyme combination*** | **Adaptors sequence (5' - 3')** | | **PCR primer(s) (5' - 3')** |
| --- | --- | --- | --- | --- |
|  |  | **First strand** | **Second strand** |  |
| #1 | PstI + AluI | CACGATGGATCCAGTGCA | CTGGATCCATCGTGCA | GATGGATCCAGTGCAG |
| #2 | PstI + BanII | Same as above | Same as above | Same as above |
| #3 | PstI + BsoBI | Same as above | Same as above | Same as above |
| #4 | PstI + BstNI | Same as above | Same as above | Same as above |
| #5 | PstI + MseI | Same as above | Same as above | Same as above |
| #6 | PstI + RsaI | Same as above | Same as above | Same as above |
| #7 | Pst + TaqI | Same as above | Same as above | Same as above |
| #8 | PstI + Tsp509I | Same as above | Same as above | Same as above |
