# Supplementary figures and images for: A MITE-based genotyping method to reveal hundreds of DNA polymorphisms in an animal genome after a few generations of artificial selection
Source: BMC Genomics. 2008 Oct 6;9:459. doi: 10.1186/1471-2164-9-459 (PMC2579443; doi:10.1186/1471-2164-9-459)

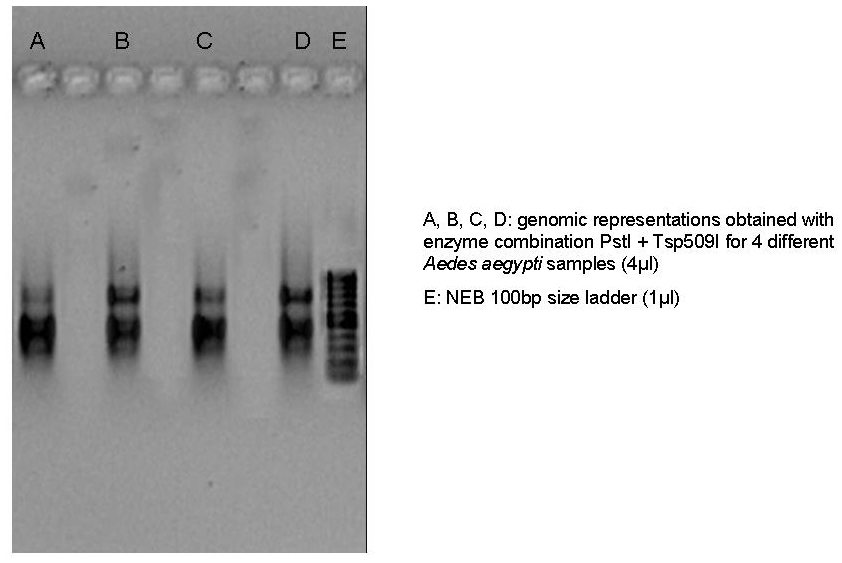

Supplement: Additional file 2 — Example of a poor-quality genomic representation obtained with enzyme combination PstI + Tsp509I. [file 1471-2164-9-459-S2.jpeg]
